# Balancing Dependence in Everyday Life- a Qualitative Study of Older Adults and Relatives’ Experiences of the Post-Discharge Home-Based Follow-Up Visit

**DOI:** 10.5334/ijic.9822

**Published:** 2026-06-23

**Authors:** Sanne Have Beck, Astrid Janssens, Grethe Eilertsen, Karen Andersen-Ranberg, Dorthe Susanne Nielsen

**Affiliations:** 1 Geriatric Research Unit, Department of Clinical Research, University of Southern Denmark, Odense, Denmark; 2 Department of Geriatric Medicine, Odense University Hospital, Odense, Denmark; 3 COPe –Research center for Culture and Older People (vulnerability), Denmark; 4 Bioethics and Health Humanities, Julius Center for Health Sciences and Primary Care, University Medical Center Utrecht, the Netherlands; 5 Department of Public Health, User Perspective and Community-based Interventions, University of Southern Denmark; 6 Centre for Research with Patients and Relatives, Odense University Hospital, Denmark; 7 University of Exeter Medical School, United Kingdom; 8 Department of Nursing and Health Sciences, Faculty of Health and Social Sciences, University of South-Eastern Norway, 58 Grønland, Drammen 3045, Norway; 9 USN Research Group of Older Peoples’Health, University of South-Eastern Norway, 58 Grønland, Drammen 3045, Norway; 10 Danish Aging Research Centre, Department of Public Health, University of Southern Denmark, Odense, Denmark

**Keywords:** older adults, from hospital to home, relatives, practice research

## Abstract

**Introduction:**

When older adults living with multimorbidity are hospitalised, they often require the involvement of multiple stakeholders to ensure continuity of care when transitioning from hospital to home. More interventions have been examined. This study aimed to explore how older adults and their relatives, spouses, or adult children experienced a nurse-led, cross-sectoral home-based follow-up visit after hospital discharge.

**Methods:**

This study is grounded in critical psychological practice research. We conducted qualitative individual and family semi-structured interviews with 10 older adults, six relatives, three spouses, and three adult children. The interviews were conducted by telephone or face-to-face. The analysis was inspired by Braun and Clarke.

**Results:**

We identified three themes: 1) Cross-sectoral home-based follow-up visit – an experience of caring and competent nurses, 2) Navigating dependency and disruption – balancing a new situation, and 3) Relatives – caught between support and caregiver burden.

**Conclusion:**

The cross-sectoral, home-based follow-up visit was considered important by some older adults and all relatives during the transition from hospital to home, as it enhanced their sense of safety. The integration of care across healthcare settings improved cross-sectoral communication, facilitated collaboration across care settings, and ensured the secure transfer of information and mutual knowledge translation.

## Introduction

Use of primary care services increases with age. In Denmark, 12% of women and 8% of men aged 65 or older receive such assistance [1]. Older adults account for approximately 250,000 hospitalised individuals annually, totalling about 600,000 admissions [2]. The transition from hospital to home for older adults, those aged 65 years and older, is a dynamic process where changes are experienced [3,4]. The transition from hospital to home has been a focus of healthcare research for decades [5,6,7] as hospitalised older adults often live with multimorbidity [8] and require involvement of multiple stakeholders during this process. Furthermore, the older adults’ pre-hospital functional and mental status, e.g. need for a walking aid or cognitive impairment, negatively affects the functional decline during hospitalisation [9]. More studies have evaluated the effects of various transitional care interventions, with some demonstrating improvements in patient safety and satisfaction [7,10]. However, these interventions remain difficult to compare due to the use of different measurement tools [11]. Lee et al. conducted a literature review examining transitional care strategies for frail older adults found that six out of 13 studies demonstrated effectiveness in reducing hospital readmissions at six months. However, only two of 13 studies showed a positive impact on patients’ quality of life, leaving the overall effectiveness of these interventions inconclusive [12]. One study, which implemented a comprehensive continuum of care intervention, found that patients who knew whom to contact after discharge reported higher satisfaction with care [13].

More studies describe that when older adults are discharged from the hospital, the adults and their relatives experience a healthcare journey which holds misperceptions and often entails a lack of involvement during discharge and communication between hospital and primary care [6,14]. A qualitative meta-summary identified that the power relationship between healthcare professionals and older adults was unbalanced, often leading older adults to feelings of powerlessness and a lack of agency, as decisions were made without their input [14].

In Norway, older adults and their relatives report tension between being a burden and carrying one, feeling responsible for securing follow-up care themselves. Relatives often found care demands exceeded their time and resources [15]. In a scoping review, 70 studies on interprofessional teams supporting transitions of older adults from hospital to primary care were identified showing various strategies, such as ‘Interprofessional Assessment’, ‘Ensure/Promote Role Clarity’ and ‘Interprofessional Care Models that Cross the Traditional Hospital Boundaries’ to support older adults in the transition [16]. In a Danish context, a study on interprofessional teams supporting transitions of older adults from hospital to a skilled nursing facility showed a reduction in readmission rates by 28% [17]. Furthermore, the involved healthcare professionals experienced interprofessional teamwork through relational coordination and shared goals for the patient [18]. At the same time, several studies have examined how transitional care interventions impact, e.g., readmission rates [11,12], more knowledge is needed about how these transitional care interventions are experienced by patients and their relatives [7,19].

This study aimed to explore how older adults and their relatives, spouses or adult children experienced a nurse-led, cross-sectoral home-based follow-up visit after hospital discharge using a practice research approach.

## Methods

### Design

Our study is the qualitative part of a larger Randomised Controlled Trial, testing the impacts and outcomes of a cross-sectoral home-based follow-up visit [20]. Our study employed a qualitative, practice research approach using semi-structured interviews [21,22] supplemented by field notes taken during the interviews [23,24] conducted within the context of the adult’s home.

We used critical psychology practice research to explore older adults and their relatives’ experiences with receiving a cross-sectoral home-based follow-up visit in the adult’s home [25]. Critical psychology is rooted in historical dialectical materialism, emphasising the older person’s dynamic interaction and participation in various social contexts [26,27]. In our study, we specifically focused on their interaction with nurses during the cross-sectoral home-based follow-up visit. To understand the experiences and perspectives of the older person, we explored their everyday life *conditions* within these diverse contexts. Conditions can represent both possibilities and limitations for the older person and their relatives, influencing how they create meaning and rationalise their actions and choices in their conduct of everyday life [26]. The conduct of everyday life is understood as an everyday social practice, where routines such as getting up in the morning, personal hygiene and eating habits provide security and safety, shaping an individual’s life trajectory [25,28]. This study followed the Standards for Reporting Qualitative Research (SRQR) guidelines [29].

### Setting and follow-up visits

The Danish healthcare system is tax-funded, and most services are free of charge. The municipalities are responsible for providing home-based healthcare services, including home care and primary care nursing, while regions are responsible for managing hospitals [30]. When older adults with ongoing care needs are discharged from the hospital to home, these needs are communicated digitally to municipal primary care services, who when the older adult is at home examine and provide care. The intervention was conducted in an urban municipality and included older adults who had been hospitalised in the Geriatric Department at a University Hospital. This integrated care initiative took place in the older adult’s home two to five days after discharge. During the visit, the hospital-based nurse and primary care nurse discussed the individual older adult’s health and well-being together with the older adult and, with the older adult’s consent, their relatives. Hospital-based nurses measured vital signs such as blood pressure and oxygen levels when deemed relevant in the individual case, and conducted blood tests using point-of-care technology (POCT) [31].

When conducting our qualitative interviews we invited older adults and relatives to choose the setting for the interview offering either a phone interview or conducting the interview face-to-face in the participant’s home between three to eight days after the cross-sectoral home-based follow-up visit.

### User involvement

At the Department of Geriatric Medicine, a geriatric research user panel comprising relatives of former geriatric patients, representatives from various non-governmental organisations as well as researchers from the geriatric research unit. This research user panel was involved in the current study and was invited to develop the interview guide. The aim of the study was introduced and the group was asked: *“If we want to know something about what is important to an older adult when discharged from the hospital to their home, what should we ask about?”* The panel contributed valuable perspectives, which were incorporated into the interview guide. One example was *“How did you feel about being at home?”*. Additionally, they emphasised the importance of exploring the relatives’ perspectives thoroughly.

### Recruitment

When inviting older adults to the study we used purposive sampling [32], to ensure a diverse group of older adults according to gender, age and family background. Inclusion criteria were older adults aged 65 years or older, requiring daily personal assistance from primary healthcare providers, and cognitively able to consent to participate [20]. The older adults and their relatives, who were invited by the older adults, received information about the study in connection with the follow-up visit. The hospital-based nurses acted as gatekeepers [22], they distributed information material and invited both older adults and relatives to participate in the interview study. The hospital-based nurses asked the older adults and relatives if the first-author could contact them with further details. Some older adults preferred that the first-author contact their relatives to plan the interview.

### Participants

A total of 15 older adults were invited to participate. Five of the older adults (three men and two women) did not participate, due to readmission, being unavailable at the scheduled time and not wanting to reschedule, or because their relatives declined participation due to the older adult’s current medical conditions expressing it being too burdensome for the older adult. All invited relatives participated. In total, 10 older adults and six relatives participated in an interview (see Table 1).

**Table 1:** Characteristics of the participants. The abbreviation ID-P will be used when referring to older adults and ID-R for relatives.


ID-P	GENDER	AGE	RESIDENCE	RELATIVES	ID-R	RELATION	LIVING STATUS RELATED TO THE OLDER ADULT	INTERVIEW

1	Male	80	House with garden	Distant relatives**				At home Face-to-face

2	Male	89	House with garden	Living with wife				Phone

3	Male	80	House with garden	Living with wife 2 daughters	11	Wife	Lives with husband	At home Face-to-face

4	Female	88	Senior housing	1 daughter				At home Face-to-face

5	Female	79	Senior housing	1 son and 1 daughter				At home Face-to-face

6	Male	94	House with stairs and garden	Son	12	Son	Lives under 1 kilometre away	At home Face-to-face/ Phone

7	Male	85	House with stairs and garden	Distant relatives**				At home Face-to-face

8	Female	85	House with garden	2 sons				At home Face-to-face

9	Male	81	House with garden	Living with wife 2 children and grandchildren	16	Wife	Lives with husband	At home Face-to-face

10	Female	96	House with garden	1 son and 1 daughter and grandchildren				At home Face-to-face

					13*	Daughter	Lives 40 kilomertes away	Phone

					14*	Wife	Lives with husband	At home Face-to-face

					15*	Daughter	Lives 15 kilometres away	Phone


*Indicates relatives who participated without the older adult. **Older adults described their relatives as distant, describing that they were not involved in the older adult’s everyday lives.

In the following, the abbreviation ID-P will be used when referring to older adults and ID-R for relatives.

## Data collection

### Interviews

The interviews were planned to be conducted as separate interviews with the older adults and relatives allowing them to elaborate on their experiences and feelings [33]. Field notes regarding the setting, the atmosphere, and the conditions were written down before, during and after the interview to ensure sensitivity to context and to support the interpretation of the interviews [24]. When the older adult had confirmed interest in participating in an interview, the first-author contacted the older adult or relative by phone. During this conversation time and place for the interview were arranged. One older adult and three relatives preferred a phone interview, and for the others, the interview was conducted face-to-face in the older adult’s home [21,34,35]. The interviews were guided by a semi-structured guide [21,22] exploring older adult’s or relative’s perspectives, previous experiences, and how they reasoned their actions. While separate interviews were initially planned, relatives present in the home were included with the older adult’s and interviewer’s consent. Two interviews were conducted as family interviews, and allowed them to elaborate on their individual and shared experiences. All interviews were audio-recorded and transcribed verbatim by the first-author. The duration ranged from 35 to 90 minutes, with a mean of 45 minutes.

### Field observations

In supplementary to the interviews field observations were conducted in the older adult’s homes to contextualise the interviews [23]. Notes were taken either during or after the interview to involve the home setting, relevant artefacts, such as calendars, as well as the involvement of relatives during the interview. These observations helped contextualise the older adult’s experiences and provided deeper insight into their situations [24].

## Analysis

The condition-meaning and reasoning analysis [27,36] was used to unfold and contextualise the participants’ experiences [37] and to explore how everyday life conditions created meaning for the individual older adult or relative [28]. The analytical process was inspired by Braun and Clarke [38]. The transcribed interviews were read, and the audio recordings were listened to multiple times by the first author to ensure familiarity with the data. The analytical process began with coding condensed notes from the interviews, focusing on the older adults’ everyday life, how they created meaning, and how they reasoned their actions. These codes were supplemented with field notes, including observations of entering the home, the use of artefacts, and the older adult’s independence in daily tasks. Across interviews, codes were organised using mind maps, enabling the identification of patterns and the development of sub-themes. Through an iterative process of revisiting interviews and notes, sub-themes were refined and merged into coherent, overarching themes. The initial analysis was discussed with the last author and subsequent all authors to validate the interpretations and ensure rich descriptions of the participants’ experiences. The analytical process outlined above is illustrated in Table 2.

**Table 2:** Example of the analytic process resulting in Theme 1 ‘Cross-sectoral home-based follow-up visit – an experience of caring and competent nurses’.


DATA	SUB-THEMES	THEME

I felt they kept an eye on me (ID-P 7) It was like being at the doctor’, they asked and we talked (ID-P 6) They (the nurses) were so nice (ID-P 8)	Experienced competent nurses	Cross-sectoral home-based follow-up visit – an experience of caring and competent nurses

Test results showed no sign of infection (ID-P 9) My haemoglobin count was low, so I had to go to the geriatric outpatient clinic (ID-P 10) I still register how much I drink, showing the fluid chart (Fieldnote ID-P 4)	The cross-sectoral home-based follow-up visit was experienced as a continuation of treatment and care	

So many people come into my house every day, and I can not remember who they are or what they do (ID-P 8)	Depending on help from primary care	

When admitted to the hospital they found he had very low sodium, but now they said it was normal, which made me feel safe (ID-R 12) When she (the hospital-based nurse) entered she talked with my dad, she looked him in the eyes and waited for him to answer… I felt so safe as I experienced my responsibility was shared… by someone who knows about older people (ID-R 13)	Relatives were supported by the cross-sectoral home-based follow-up visit	


### Reflexivity

Before the study, the first-author worked as a Registered Nurse (RN) and clinical nurse specialist in the Department of Geriatric Medicine. I critically reflected on my prior position and conditions for influencing the research process and exploring the aim of this study [39]. To minimise the potential influence of being an RN, I presented myself as a PhD student and during the interview listened carefully and with curiosity as unfolding the participant’s perspectives and experiences of the cross-sectoral home-based follow-up visit. The first author had experience conducting qualitative interviews, and the last author is an experienced qualitative researcher. A reflexive process was ensured as reflections and discussed with the last author to ensure ongoing sensitivity and reflections on positioning in conducting research.

### Ethical considerations

All participants were informed in writing and verbally about the aim and method of the study when first approached by the gatekeeping hospital-based nurse and at the beginning of the interview, and written consent was obtained for all participants. During the interviews in the older adults’ homes, it became evident that the older adults involved in this project were in a vulnerable position in the first days after discharge, they were dependent on others, adapting to a new situation, and in some situations, they lacked ability to act, which impacted the interviews [24]. Entering the home and approaching the older adult’s everyday life demanded knowledge of older adults, compassion, dignity, respect and ethical considerations from initial contact, entering the home and leaving [40]. During the interviews, SHB was mindful of the older adult’s ability to remember e.g. the home-based follow-up visit and being sensitive in asking and withdrawing when experiencing a lack of ability to answer [41].

The study was conducted adhering to the Helsinki Declaration [42]. It was approved by the Ethical Committee (Projekt-ID: S-20210157) and the Data Protection Agency -Region of Southern Denmark (Journal nr.: 21/57714).

## Results

The interviews and field observations revealed that the older adults’ transition from hospital to home and the first week at home often comprised adjustments to care routines, more than usual. Some older adults had a hard time remembering and distinguishing the different healthcare professionals. However, all of the participating relatives were able to distinguish the staff related to home-based follow-up visits from other healthcare professionals visiting the home. In the interviews, all older adults focused on the transition from hospital to home and the importance of having relatives, even the older adults who described having distant relatives. The analysis resulted in the following three themes: ‘Cross-sectoral home-based follow-up visit – an experience of care and competent nurses’, ‘Navigating dependency and disruption –balancing a new situation’, and ‘Relatives – navigating involvement and consequences of dependency’. In the findings, the “cross-sectoral home-based follow-up visit” will be referred to as a “follow-up visit”.

### Theme 1 Cross-sectoral home-based follow-up visit – an experience of caring and competent nurses

The follow-up visit took place 2–5 days after discharge from the hospital. For some older adults, the follow-up visit enhanced feelings of being seen and cared for. One older adult remarked: “I felt they kept an eye on me” (ID-P 7). During the interviews, older adults and relatives frequently talked about the nurses attending the follow-up visit as highly competent in assessing and identifying changes and at the same time kind and smiling. Relatives valued the empathetic and engaged approach of the nurses. One daughter described her experience:

“When she (the hospital-based nurse) entered, she talked with my dad, she looked him in the eyes and waited for him to answer… I felt so safe as I experienced that my responsibility was shared… by someone who knows about older people” (ID-R 13).

However, not all older adults were able to distinguish the healthcare professionals involved in the follow-up visit from other help provided, as they described the presence of multiple healthcare professionals. One older adult noted:

“So many people come into my house every day, and I can not remember who they are or what they do, but they are all very kind” (ID-P 8).

More relatives described the follow-up visit as an important support, supplementing the hospital discharge process. During this visit, important topics were addressed, including the older adult’s treatment, well-being, and everyday challenges. For instance, the ability of hospital-based nurses to collect blood samples and share results on the spot created a sense of reassurance for both relatives and older adults:

“Test results showed no sign of infection” (ID-P 9).

Receiving prompt test results alleviated older adults’ worries and contributed to a sense of safety and reassurance, reinforcing their perception of being monitored and cared for. Relatives felt reassured about the older adult’s condition when vital signs or test results were presented:

“When admitted to the hospital, they found he (father) had very low sodium, but now they said it was normal, which made me feel safe” (ID-R 12).

More relatives underlined the importance of the nurse being able to collect blood samples as a continuity of treatment from the hospital. For some, the results ensured the treatment was successful, for other older adults, the test results were abnormal. Either way, the immediacy of the test results offered assuredness for both older adults and relatives. One older adult said:

“My haemoglobin count was low, so I had to go to the geriatric outpatient clinic, …but we made a plan” (ID-P 10).

When needed, the hospital-based nurse was able to plan further treatment right away; this improved the older adult’s experience of being guided and involved in the planning. Relatives appreciated the opportunity to discuss signs of potential illness and the older adult’s progress with both hospital-based and primary care nurses. The collaboration between the hospital-based nurse and primary care nurse provided coherence for the relatives, they experienced that information was exchanged between the nurses. Furthermore, older adults and their relatives felt that the nurses’ different knowledge and expertise created a shared understanding of the older adult’s situation and felt they had someone to rely on in the future.

### Theme 2 Navigating dependency and disruption – balancing a new situation

During the interviews, all older adults shared experiences about their hospital stay and their discharge home, as well as being in a new situation. Some older adults described feeling ready to be discharged: they described feeling better and also had a feeling of physical recovery, feeling well enough to go home. Other older adults shared that they felt insecure in the days leading up to the discharge. Poor communication regarding the discharge date and loose ends about their medical results left them feeling worried. One older adult recalled the days before discharge from the hospital:

“They kept saying that I was to go home, but they kept postponing the actual date. They needed more tests… I was sick and tired of it” (ID-P 2).

These older adults felt dependent on healthcare professionals for information while simultaneously experiencing a lack of clear communication, which led to insecurity and sadness. These emotions persisted during the transition home, affecting both older adults and their relatives and diminishing their trust in primary healthcare providers. One older adult, who had been admitted multiple times reflected on his experiences with this last hospital admission:

“I had never been admitted to the hospital before I was 85 years old. This year I have been admitted multiple times.… But for the first time, I agreed to be discharged. I felt better and felt that I was able to manage (being at home)” (ID-P 6).

The primary healthcare providers met the older adult’s physical dependence which resulted in frequent visits and altered the dynamics of the older adult’s everyday life. While grateful for the assistance, older adults struggled to adjust to the help provided. One older adult described this disturbance:

“People fly in and out of our home, and we normally live a quiet life, only our family is here but now people come and go, and it feels like a train station” (ID-P 3).

More older adults described that the help offered did not align with their daily routines. Older adults reacted differently to the help offered by the primary healthcare providers, some accepted, others negotiated and some declined the help.

Older adults who accepted the help described the help received as violating their daily routines, e.g. around their meals:

“They are supposed to help with breakfast, but more times I have made it myself, I usually get up at 6 am, and sometimes they are here by 8 am other days at 10 am” (ID-P 5).

“They sometimes serve breakfast at 10 o’clock and then serve lunch at 11.15, but I have not worked up an appetite in such a short time” (ID-P 6).

This resulted in a trade-off between accepting the help offered and disrupting daily routines and doing things themself. The older adults described that they did what they were able to do themselves e.g. around the meal, yet, this had consequences such as not being able to gain weight. Some older adults felt unsafe living alone, doubting whether primary healthcare providers would notice or respond if their condition worsened.

During the analysis, we found that for older adults receiving help from primary care, the ability to actively negotiate the type and extent of assistance was an important factor in their sense of autonomy and well-being. This negotiation was closely tied to their interactions with primary healthcare providers. One older adult shared:

“I want to do the things I was able to do by myself. The primary care nurse offered to help dose my medicine, but I said no thank you and argued why I would do that myself. She thought it sounded reasonable and asked if she could do anything else for me” (ID-P 3).

Hence, being recognised as an individual with unique preferences and values fostered a sense of security and trust in the care provided.

Older adults who were not satisfied with primary healthcare providers’ services declined some of the help as it was not tailored to e.g., their circadian rhythm and life together. This decline in help made the older adults more dependent on help from their relatives often spouses. One couple said:

P: “sometimes they (primary healthcare providers) came at 7 PM when we were watching a movie”

R: “but more times we had to wait up, so now I help with his catheter at night…” (ID-P 9 and ID-R 16).

### Theme 3 Relatives – caught between support and caregiver burden

All older adults emphasised the emotional and practical reassurance of having their relatives around when they were discharged from the hospital. Older adults who had previous experience with hospital stays worked towards a “safe transition to home”; for some this meant relatives moving in with the older adult, and being at the older adult’s home or living nearby reassured both the older adult and the relative feeling safe. One older adult described her first evening at home, highlighting the importance of her daughter’s support:

“She cooked me dinner and helped me to bed. I felt safe. I felt like everything would be fine” (ID-P 4).

For another older adult, the longing to return home to his wife after a two-week hospitalisation, due to a bowel infection, led to acceptance of being discharged. He reflected on the time before discharge from the hospital:

“I thought “I would love to go home, I didn’t think of what a burden I would be on her (wife)”. It was too early” (ID-P 3).

His wife endured the difficulties of the early discharge:

“Yes, it was… It was so fine that homecare was established, but he could not wait for them to arrive when he needed to go to the toilet. You have to be there. … The weekend was hard, he should not have been discharged until Monday. But we made it” (ID-R 11).

Relatives were essential to older adults’ lives following hospital discharge, as they consistently navigated diverse tasks to ensure continuity of care and provide critical support. Spouses were particularly engaged in physical care tasks, such as assisting with mobility, toileting, and other personal needs. For some older adults, this dependence came at the expense of relying on relatives to step in, particularly for personal care tasks.

As one couple explained:

R:… “I think what hurt (older adult’s name) the most is that he can’t do anything anymore”

P: “I can’t”

R: “But we have to take it as it comes”

P: “I have thought about how long (wife’s name) can handle it all (crying)”

R: “I believe I can, but I am easily moved” (ID-P 9 and ID-R 16).

Having lived a long life together provided spouses with deep insights into their partners’ strengths and vulnerabilities. However, adapting to these new caregiving demands required significant adjustments. One self-employed spouse described how she managed her husband’s care alongside her work responsibilities:

“I need to be flexible every time my husband is discharged, with no say in the date or time he returns home. It leaves me with no choice but to cancel or reschedule meetings… At home, it doesn’t end—when the primary healthcare providers do not provide scheduled help, I’m the one filling in… My job is important because otherwise, I’d be his full-time nurse” (ID-R 14).

For adult children caring for their parents, involvement in the caring duties varied widely depending on physical, psychological, and social circumstances. Proximity was important, as those living nearby were able to visit often, while others faced logistical challenges. One daughter shared:

“I have to take a taxi every time I visit her, it gets rather expensive, so we talk on the phone every day at 7.00 AM, so I know that she is all right” (ID-R 15).

More of the adult children had prior experiences with their parents being discharged from the hospital and they elaborated on strategies they now used when their parent was discharged. One daughter recalled an incident where her father had been left unattended post-discharge, and described her decision to always be present when he returned home:

“From that day on, I always took time off from work to be at his house when he was discharged” (ID-R 13).

In some cases, adult children moved in temporarily with their parents to ensure a safe transition, feeling a deep sense of responsibility. This responsibility extended beyond the initial days, as adult children managed various aspects of their parents’ care, such as monitoring health conditions, scheduling doctor visits, or ensuring medical aids (for example cream, and incontinence pads) were present in the house at all times. Despite these time-consuming demands, they remained committed:

“Please don’t hear that I think it is too much, it is a lot, but I also want to be there for them” (ID-R 13).

“Engaging almost demands that you are a senior citizen” (ID-R 12).

Relatives mentioned that their involvement was vital to ensure a safe transition and ensure healthcare professionals adapted their care at home right after discharge from the hospital mediating and ensuring help. They emphasised that these challenges were not due to individual healthcare professionals, but organisational challenges at the hospital and home.

Older adults who were dependent on help from primary healthcare providers, but without close relatives, added an additional layer of complexity to the help required. One older adult reflected on his isolation:

“I have been a hermit/a recluse all my life, and that has worked perfectly, but maybe not so much growing old. Now I think that I might have to move into some sheltered housing, as I can’t do everything myself, even though I would rather stay in my house” (ID-P 7).

Another older adult, widowed six years earlier, described the profound loneliness of living without close family or social connections. While he had successfully stopped drinking after a period of alcohol abuse, the emotional toll of isolation remained a significant challenge:

“I try to maintain daily routines, but the feeling of loneliness is hard” (ID-P 1).

For those older adults without close relatives, the transition and receiving help post-discharge underscored the difficulties of maintaining independence and a sense of belonging and faced both physical, psychological and social vulnerabilities.

## Discussion

In this study, we aimed to explore how older adults and their relatives, spouses or adult children experienced a nurse-led, cross-sectoral home-based follow-up visit after hospital discharge using a practice research approach. We found that some older adults and all relatives found the home-based follow-up visit valuable in creating continuity of care. Also, older adults and relatives appreciated the home-based follow-up visit as they felt secure when the blood test and vital signs showed progress in the older adult’s recovery. Furthermore, they valued integrated care by having a nurse from the geriatric department – who had extensive knowledge about older people, diseases and older adult’s hospital stays meeting the primary care nurse securing mutual information and knowledge translation. This follow-up visit assured the relative had someone to share the feeling of responsibility with at home. In a study by Berglund et al. [13] offering care-planning meetings a few days after discharge, with the participation of a case manager, an interprofessional team from the municipality, and relatives, a high proportion of participants responded ‘agree completely’ to the item about the value of conducting the care-planning meeting at home [13]. A newly conducted scoping review including 23 studies of interventions in transitions between healthcare settings, of which five used a qualitative method, showed interventions can have a positive effect in navigating between healthcare settings as older adults express better experiences feeling a sense of support [43]. In line with these findings, our study underlined that hospital-based nurses and primary care nurses providing follow-up visits created a sense of continuity of care between hospital and home.

We found that many older adults experienced primary healthcare support as misaligned with their daily routines. After hospital discharge, declines in functional status often coincided with a transition period in which older adults had to adapt to providers’ various schedules. Responses varied: some accepted, others negotiated, and some declined the help. Frequent visits sometimes disrupted routines and daily life, and more older adults reported that care was not always provided at times that met their needs, such as bedtime or mealtimes. For most older adults, these familiar doings are established routines in their conduct of everyday life as these tasks might not have been questioned for years, but part of their circadian rhythm. Also, routines represent personal preferences for ways of doing things and come with a degree of agency and autonomy [25]. We found that those having relatives were in a stronger situation and able to negotiate with primary healthcare providers, resulting in more individualised and tailored care. Other older adults with relatives, declined the help offered, and these older adults became dependent on their relatives to provide, e.g. personal care. The conduct of everyday life is dynamic and must constantly be protected and negotiated to fit into organisations/contexts, e.g. primary healthcare providers’ time, which can potentially threaten the feeling of control in older adults’ lives [25]. Being dependent on relatives and healthcare professionals can threaten the older adult’s autonomy and increase the older adult’s experience of vulnerability. Similar findings are also identified in a meta-synthesis of 53 studies, where several studies found that patients were dependent during the hospital stay and identified the importance of older adults regaining individuality and independence when at home after hospital discharge. Furthermore, it was found that some older adults missed their daily routines during their hospital stay, and thereby preferred being at home [44]. In our study, we found that more older adults struggled to maintain daily routines at home after discharge from the hospital.

In our study, we found that the transition from hospital to home was either experienced by the older adults as premature or as feeling well enough to go home and affected the time at home post-discharge. Premature discharge is also found in the study by Skovgaard et al [45] describing the negotiation between healthcare professionals avoiding obstructions in flow at the hospital and sorting out tasks to be handled by others, labelling the older adult as “fully treated” [45]. This experience affected the days after hospital discharge at home where primary healthcare providers were ready to provide care and help. Perspectives of both older adults and their relatives on transitioning from hospital to home have been explored in a meta-ethnography conducted in 2023 including 10 studies, holding six studies from Scandinavian countries [46]. They found that older adults and relatives either experienced being seen as objects and excluded from the transition process or included in the transition process, where they were informed and actively involved in the transition process. The feeling of being excluded from the transition process caused suffering. Older adults and relatives who felt involved in the transition process experienced dialogue and information as factors as they felt prepared for every phase of the process [46].

For older adults who described living with distant relatives, increased the complexity as they wished to stay in their homes, but did not experience the help provided covering their practical, psychological or social needs. They feared the need to move, struggled with loneliness and experienced a lack of ability to maintain routines, threatening their autonomy [25]. Experience of reduced autonomy and increased dependence in transitional care is also found in studies in the systematic review; having a relative involved in the transition was, in some cases, experienced as decisions were made for them. Other studies found that the relatives were supporting the older adults’ involvement and participation [47]. Older adults living with distant relatives might be in need of longer transitional care interventions ensuring the care provided will fit their daily routines and complement their multifaceted needs at home post-discharge ensuring a healthy transition [4].

We planned to interview older adults and relatives separately to ensure their ability to express their feelings on sensitive topics [33], but we experienced when conducting the interviews in the older adult’s home that they invited their spouse into the interview. We had ethical reflections around this and chose to follow the wishes of the older adults and relatives and in some cases conducted the interview as family interviews. The family interviews gave valuable insights into a long life lived together and they were able to share and talk about sensitive topics. Both interviews and field observations provided insights into an interview situation where feelings were shared and allowed for both laughs and crying and first-author was not followed out the door for a further talk or contacted after the interviews. Discussions on professional background in healthcare and knowledge of geriatric nursing might have influenced the sensitivity during the interviews [48].

### Strengths and limitations of the study

This study had several strengths. The qualitative study allowed us to explore both older adults’ and relatives’ first-person experiences with the cross-sectoral home-based follow-up visit. The interviews were guided by a semi-structured interview guide, so the topic of interest was framed, but at the same time allowed for the participants to elaborate on topics that occurred during the interview. Another strength was that the participants were not excluded when preferring phone interviews over face-to-face interviews, allowing the participant to decide what was best suited at the time of the interview. However, conducting interviews over the phone could be a limitation lacking the ability to be sensitive to the participant’s body language. It could be a limitation that the hospital-based nurses were gatekeepers in the inclusion of participants, as they could have invited only participants who seemed satisfied with the home-based follow-up visit. We know less of the older adults, whose relatives declined their participation in the interview. Having relatives as gatekeepers might have limited our study population, and we might have led to the exclusion of vulnerable older adults leading to inequality in research [49]. Nevertheless, we experienced a willingness from the older adults and their relatives to share both positive and negative experiences.

## Conclusion and implication for practice

The cross-sectoral home-based follow-up visit, was by some older adults and all relatives experienced as important in the transition from hospital to home as it enhanced their feeling of safety. The integration of care across healthcare settings provided insight into how hospital-based and primary care nurses coordinate responsibilities, communicate clinical information, and support older adults, thereby contributing to safer transitions from hospital to home. At the hospital, older adults need to feel ready for hospital discharge to promote a healthy transition. Furthermore, primary healthcare providers need to adjust their provided help to promote older adults’ routines and conduct of everyday life. The cross-sectoral home-based follow-up visit enhanced the cross-sectoral communication and provided some older adults and all relatives with an experience of collaboration from hospital to home, securing information and mutual knowledge translation. Older adults with distant or no relatives might be in a more vulnerable position and need a heightened awareness among healthcare professionals when discharged from the hospital and at home post-discharge. The hospital-based nurses’ ability to use POCT in the older adult’s home was experienced as convenient and along with their extensive knowledge of geriatric nursing might be a place for further initiatives.
